# Promotional effect of magnesium oxide for a stable nickel-based catalyst in dry reforming of methane

**DOI:** 10.1038/s41598-020-70930-1

**Published:** 2020-08-17

**Authors:** Ahmed S. Al-Fatesh, Rawesh Kumar, Anis H. Fakeeha, Samsudeen O. Kasim, Jyoti Khatri, Ahmed A. Ibrahim, Rasheed Arasheed, Muhamad Alabdulsalam, Mahmud S. Lanre, Ahmed I. Osman, Ahmed E. Abasaeed, Abdulaziz Bagabas

**Affiliations:** 1 Chemical Engineering Department, College of Engineering, King Saud University, P.O. Box 800, Riyadh, 11421 Saudi Arabia; 2 Sankalchand Patel University, Visnagar, Gujarat 384315 India; 3 National Petrochemical Technology Center (NPTC), Materials Science Research Institute (MSRI), King Abdulaziz City for Science and Technology, P.O. Box 6086, Riyadh, 11442 Saudi Arabia; 4 School of Chemistry and Chemical Engineering, Queen’s University Belfast, Belfast, BT9 5AG Northern Ireland UK

**Keywords:** Chemical engineering, Environmental sciences, Chemistry

## Abstract

The generation of synthesis gas (hydrogen and carbon monoxide mixture) from two global warming gases of carbon dioxide and methane via dry reforming is environmentally crucial and for the chemical industry as well. Herein, magnesium-promoted NiO supported on mesoporous zirconia, 5Ni/xMg–ZrO_2_ (x = 0, 3, 5, 7 wt%) were prepared by wet impregnation method and then were tested for syngas production via dry reforming of methane. The reaction temperature at 800 °C was found more catalytically active than that at 700 °C due to the endothermic feature of reaction which promotes efficient CH_4_ catalytic decomposition over Ni and Ni–Zr interface as confirmed by CH_4_–TSPR experiment. NiO–MgO solid solution interacted with ZrO_2_ support was found crucial and the reason for high CH_4_ and CO_2_ conversions. The highest catalyst stability of the 5Ni/3Mg–ZrO_2_ catalyst was explained by the ability of CO_2_ to partially oxidize the carbon deposit over the surface of the catalyst. A mole ratio of hydrogen to carbon monoxide near unity (H_2_/CO ~ 1) was obtained over 5Ni/ZrO_2_ and 5Ni/5Mg–ZrO_2_, implying the important role of basic sites. Our approach opens doors for designing cheap and stable dry reforming catalysts from two potent greenhouse gases which could be of great interest for many industrial applications, including syngas production and other value-added chemicals.

## Introduction

The production of syngas (a mixture of H_2_ and CO) through dry reforming of methane is an excellent strategy to reduce the global warming effects of carbon dioxide (CO_2_) and methane (CH_4_). Noble metals such as palladium (Pd), platinum (Pt), and ruthenium (Ru) have been used for this purpose, but costly precursors and instability of catalyst, at high reaction temperature around 800 °C, have limited their application^1^. On the other hand, cost-effective nickel (Ni) metal, supported on an appropriate supports such as alumina^2^, mesoporous silicates^3–7^, and zirconia^8–10^, has been found to withstand at this reaction temperature (800 °C). In this context, many researchers have followed the surface modification methodology to optimise the catalyst performance because Ni-based catalyst is also prone to deactivation. The first series of modifications were carried out over alumina supports with K^11,12^, Mg, Ca, Ba, Sr ^13–16^, Y^17^, La^18^, Ce^19^, K-Ce^20^, Ti^21^, Zr^22,23^, Mo, W^21^, Mn^24^, Co & Cu^25^, Zn^26^, B^27^, Si^21^, and Sn^14^. Due to the extended pore network (from micro to meso) and easy pore tunable synthetic methodology of silicates, silica support is preferable over alumina support^28^. Therefore, the second series of modifications were carried out over mesoporous silicates supports with Li^29^, K^30^, Mg^31,32^, Ca^30^, Ba^33^, La^34^, Ce^30,35,36^, Zr^37,38^, Mn^38^, Co^39,40^, Cu^41,42^, Zn^40^, Al^43^ and Sn^44^. However, neither alumina nor silica supports can utilize their lattice oxygen during carbon deposit oxidation at the surface, but zirconia support does and is thus are used as oxygen carrier materials. Zirconia support is characterized by its thermal stability, an expanded network, and the ability to utilize its mobile oxygen during the surface reaction^45^. The third series of modifications were carried out over zirconia supports with K^46^, Mg^47–50^, K-MgO^51^, Ca^52,53^, La^54,55^, and Ce^56–58^. A brief literature survey of promoter/modifiers that were utilized over Ni-doped different supports is given in Table 1.

**Table 1:** A brief literature survey of promoter/modifier Ni-doped different supports.

Active metal	Modified/promoter		Support: Al_2_O_3_	Support: SiO_2_	Support: ZrO_2_
Ni	Group I	Li		^29^	
Ni		K	^11,12^	^30^	^46^
Ni	Group II	Mg	^13–16^	^31,32^	^47–51^
Ni		Ca	^13–16^	^30^	^52,53^
Ni		Sr	^13–16^		
Ni		Ba	^13–16^	^33^	
Ni	Group III	Y	^17^		
Ni		La	^18^	^34^	^54,55^
Ni		Ce	^19,20^	^30,35,36^	^56–58^
Ni	Group IV	Ti	^21^		
Ni		Zr	^22,23^	^37,38^	–
Ni	Group VI	Mo	^21^		
Ni		W	^21^		
Ni	Group VII	Mn	^24^	^38^	
Ni	Group IX	Co	^25^	^39,40^	
Ni	Group XI	Cu	^25^	^41,42^	
Ni	Group XII	Zn	^26^	^40^	
Ni	Group XIII	B	^27^		
Ni		Al	–	^43^	
Ni	Group XIV	Si	^21^	–	
Ni		Sn	^14^	^44^	

Use of strong solid base as CaO and MgO showed significant improvement and facilitated the catalytic performance with prompt adsorption of slightly acidic CO_2_ during dry reforming reaction over Ni-based catalysts. CaO coprecipitated Ni supported ZrO_2_ was well studied for different types of carbon species deposited over the catalyst surface during dry reforming of methane^59^. MgO modified Ni system is known for outstanding coking tolerance^60^. Chunwen Sun et al. showed that MgO modification might help to stabilize the lattice oxygen sites of NiO which effectively decrease the carbon deposition or graphitic layer formation^61^. Garcia et al. prepared the Ni/MgO–ZrO_2_–MgO (MgO loading in the range of 1–5 wt%) catalysts by co-precipitation method and found out that the CO_2_ and CH_4_ conversions were less than 35%^47^. Asencios and Assaf loaded Ni and Mg with different ratios on zirconia support by wet impregnation method and found out that catalyst with 20 wt% Ni and 20 mol% Mg has the best performance, where the activity was less than 80% in the oxidative reforming of methane^49^. Most of the research outputs in the literature used high loading of Ni or MgO (as high as 35 mol%) for the dry reforming reaction as Montoya et al. via sol–gel method^56^ and Titus et al. via melt impregnation^50^.

Herein, we prepared four catalysts via incipient wetness impregnation method, where the support was mesoporous zirconia, nickel as the active catalyst, and magnesium oxide as a promoter. We varied the amount of magnesium oxide to find its optimum loading for the best catalytic performance. Furthermore, we optimised the performance by varying reaction temperature. Catalysts were characterized by TGA, N_2_ physisorption, XRD, H_2_-TPR, and CO_2_-TPD. To understand the surface chemistry in optimizing the catalytic activity along with the stability of the modified catalyst, CO_2_-TPD, H_2_-TPD and O_2_-TPO of spent catalyst were also performed. A very fine-tuning, among catalytic activity and characterization results were performed; this will help to better understand the surface behaviour towards syngas production from dry reforming of methane.

## Results and discussion

**Figure 1:**
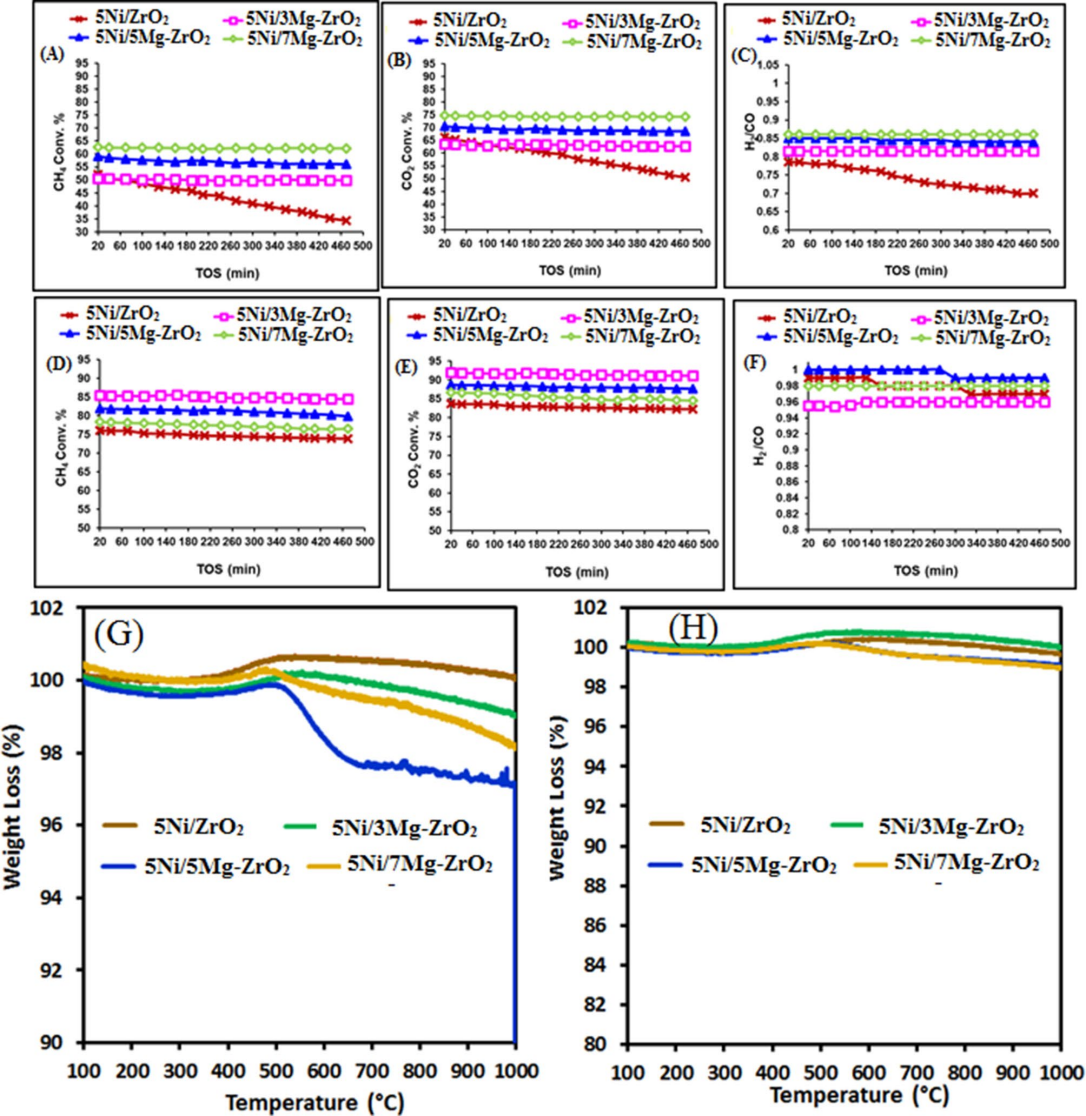
Catalytic activity profiles for methane dry reforming over various catalysts (**A**–**F**); (**A**) CH_4_ conversion at 700 °C reaction temperature, (**B**) CO_2_ conversion at 700 °C reaction temperature, (**C**) H_2_/CO mole ratio at 700 °C reaction temperature, (**D**) CH_4_ conversion at 800 °C reaction temperature, (**E**) CO_2_ conversion at 800 °C reaction temperature, (**F**) H_2_/CO mole ratio at 800 °C along with TGA curves of spent catalysts, (**G**) TGA results of spent catalyst carried out at 700 °C reaction temperature, (**H**) TGA results of spent catalysts carried out at 800 °C reaction temperature.

The catalytic activity of 5NixMgZr catalysts (x = 0, 3, 5, 7) in terms of CH_4_ conversion, CO_2_ conversion, and H_2_/CO mole ratio at 700 °C are shown in Fig. 1(A–C) and at 800 °C are shown in Fig. 1(D–F). The TGA results of spent catalysts are shown in Fig. 1(G,H), respectively. It is worth noting that without magnesium oxide modification, catalyst 5Ni/ZrO_2_ shows lower catalytic activity than that of magnesium oxide modified catalyst in all cases. At the reaction temperature of 700 °C, 5Ni/xMg–ZrO_2_ catalysts showed approximately 50–60% CH_4_ conversion and 65–75% CO_2_ conversion which were comparable to those in the recent publications^10,47,49,54,62,63^. The TGA results of these spent catalysts also showed carbon deposition. Interestingly, when the reaction temperature was set at 800 °C, it gave a stable performance with constant high conversion up to 500 min in the time-on-stream test (TOS) and no noticeable carbon deposition. Over 5Ni/3Mg–ZrO_2_ catalyst, 85% CH_4_ conversion, 92% CO_2_ conversion and 0.94 H_2_/CO ratios were achieved constantly up to 500 min in the TOS. On the target of H_2_/CO = 1, the performance of the 5Ni/5Mg–ZrO_2_ catalyst was found to be the best as it showed 82% CH_4_ conversion, 87% CO_2_ conversion. The 5Ni/7Mg–ZrO_2_ catalyst performance was a little bit lower than that of 5Ni/5Mg–ZrO_2_ (78% CH_4_ conversion, 86% CO_2_ conversion and H_2_/CO = 0.98). Whether to check the thermal decomposition of CH_4_ at 800 °C, reaction without catalyst was carried out with substrate CH_4_. It gave 3% CH_4_ conversion with 1.8% H_2_ yield in 3 h time on stream. Further again, a blank reaction was carried out with substrates CH_4_ and CO_2_ together at 800 °C. It resulted in 1.6% CH_4_ conversion, 3.6% CO_2_ conversion, H_2_ yield 0.63%, CO yield 4.25% and H_2_/CO = 0.14. As our catalytic systems are highly active towards DRM, so the thermal decomposition of CH_4_ as an intermediate step in DRM could be neglected.

To understand the surface behaviour of the DRM reaction, we characterised the catalyst thoroughly and discussed the characterization results herein. The surface area analysis indicated that after the addition of MgO, type IV adsorption–desorption curve with H1 hysteresis loop (Figure S1) was built up. It indicates the narrow distribution of mesopores.

**Figure 2:**
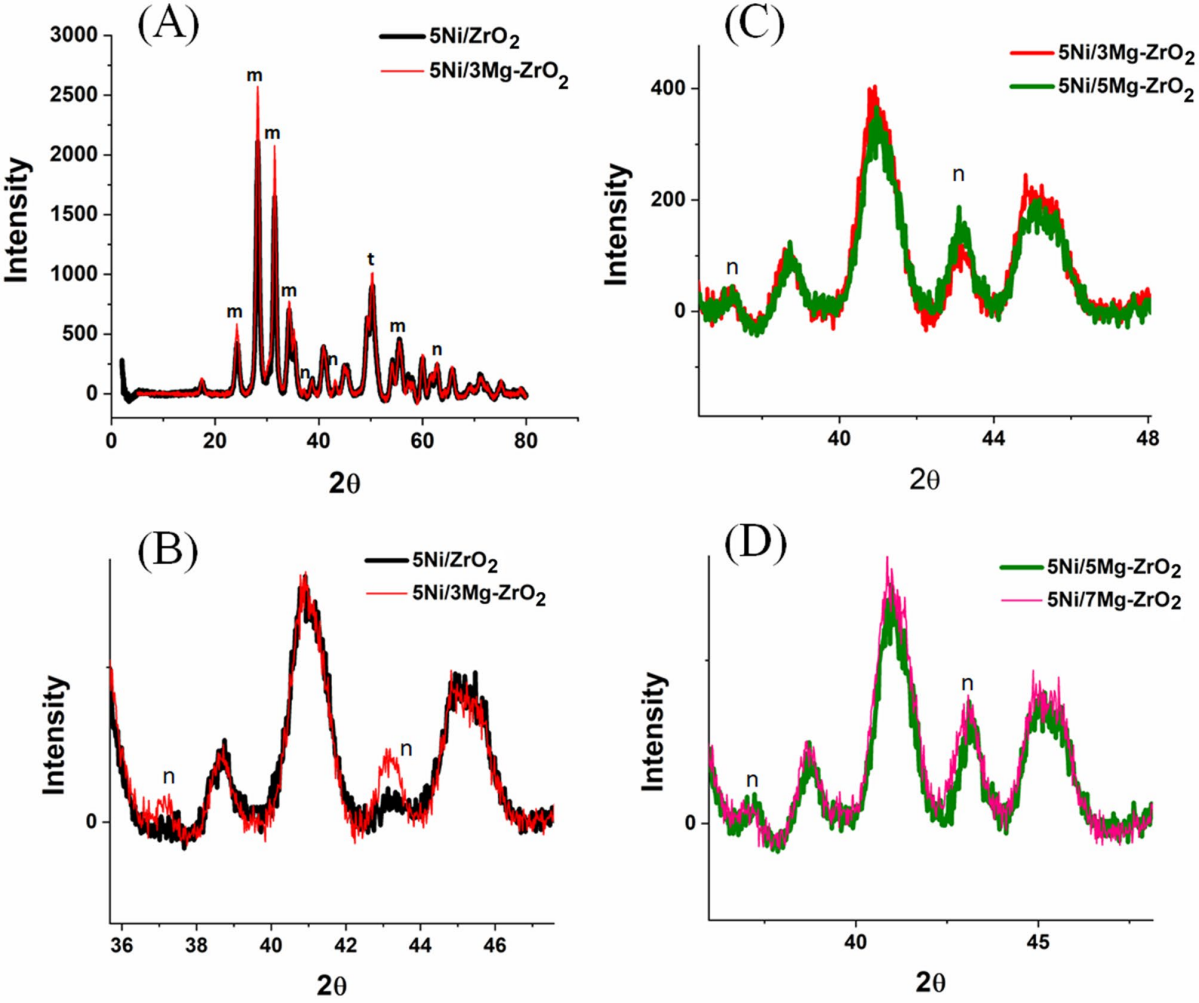
XRD of catalyst samples: m = monoclinic zirconia (m-ZrO_2_), t = tetragonal zirconia (t-ZrO_2_), n = NiO.

XRD patterns of 5NixMgZr catalysts (x = 0, 3, 5, 7) are shown in Fig. 2(A–D). The diffraction lines at 2θ = 24.2°, 28.34°, 31.45°, 34.2°, and 55.4° were attributed to the monoclinic zirconia (m-ZrO_2_) whereas diffraction lines at 2θ = 30.48° and 50.24° were attributed to tetragonal zirconia (t-ZrO_2_). Cubic nickel oxide showed diffraction lines at 2θ = 37.2°, 43.28° and 62.9° for (111), (200) and (220) crystallographic planes, respectively. After the addition of basic promoter 3 wt% MgO, the crystalline peak intensity of ZrO_2_ remarkably increased as well as the selected plane of NiO (200) about 43.28° bragg angle also intensified and shifted to the lower angle 43.12°. It indicated the rapid growth of NiO-MgO solid solution^50^ after addition of MgO. Further addition of MgO did not show such a rapid rise of NiO-MgO solid solution.

**Figure 3:**
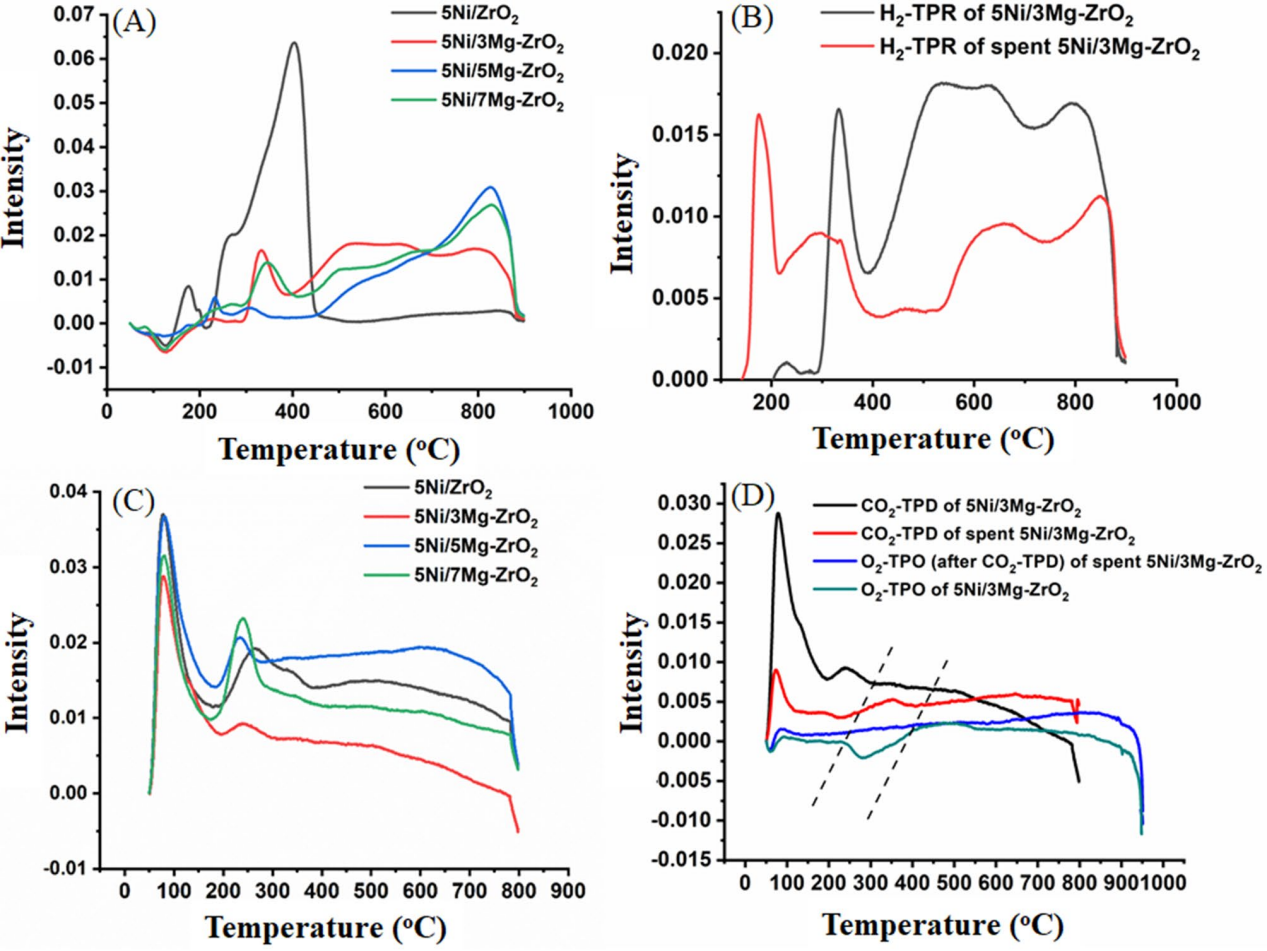
(**A**) The H_2_-TPR profile of 5Ni/xMg-ZrO_2_, (**B**) the H_2_-TPR profile of fresh and spent 5Ni/3Mg-ZrO_2,_ (**C**) the CO_2_-TPD surface reduction profile of 5Ni/xMg-ZrO_2_, (**D**) CO_2_-TPD and O_2_-TPO TPD profile of fresh and spent 5Ni/3Mg-ZrO_2_ catalyst.

The H_2_-TPR surface reduction profiles of fresh 5Ni/xMg–ZrO_2_ catalysts are shown in Fig. 3A. 5Ni/ZrO_2_ has one small reduction peak in the temperature range of 140–200 °C that attributed to the free NiO species, a shoulder reduction peak at the temperature range of 200–300 °C for “NiO weakly interacted with ZrO_2_ support” and a strong peak at 300–450 °C for “NiO that interacted strongly with ZrO_2_ support”. After the addition of 3.0 wt% MgO, these three peaks diminished and reduction peaks in the intermediate and high-temperature ranges appeared. The high reduction temperature for MgO modified samples could be correlated to the high inherent stability expected for NiO–MgO-solid solution with respect to pure NiO. From the XRD results, also after MgO modification, NiO–MgO-solid solution was found^50^. The intermediate temperature reduction peak in the range of 450–700 °C could be attributed to “NiO–MgO-solid solution weakly interacted with ZrO_2_ support” whereas high-temperature reduction peak in the range of 700–900 °C could be claimed to “NiO–MgO-solid solution strongly interacted with ZrO_2_ support”. As MgO loading was increased from 3.0 wt% to 5.0 wt%, the TCD signal intensity of the intermediate temperature reduction peak was decreased and high-temperature reduction peak was increased. These observations indicated that a higher amount of “NiO-MgO-solid solution strongly interacted on ZrO_2_” was present in 5Ni/5Mg–ZrO_2_ than 5Ni/3Mg–ZrO_2_, thus 5 wt% MgO was the optimum loading. At 7 wt% MgO loading, both types of high-temperature peaks were suppressed in comparison to those for 5Ni/5Mg-ZrO_2_. The H_2_-TPR surface reduction profile of spent 5Ni/3Mg–ZrO_2_ is shown in Fig. 3B. It showed that TPR peaks in the intermediate and high-temperature regions had got suppressed. Also, it was noticeable that a lower reduction temperature peak (0–400 °C) remained preserved as well as shifted to a lower temperature. The H_2_-TPR surface reduction profile of spent 5Ni/5Mg–ZrO_2_ indicated the suppression and shifting of high-temperature region peaks to intermediate temperature regions (Fig. S2). These observations indicated that NiO supported on ZrO_2_ was less involved whereas “NiO-MgO-solid solution interacted with ZrO_2_ support” are significantly involved in DRM. Apart from that, the elimination of carbon deposit by hydrogen gas during methane gasification reaction (C + 2H_2_ → CH_4_) over spent catalyst system was also possible^64^.

The CO_2_-TPD profiles of 5Ni/xMg–ZrO_2_ are shown in Fig. 3C. Without magnesium oxide modification, the catalyst showed a sharp peak at lower temperature (weak basic sites) region and in intermediate temperature (medium basic sites) regions, but a broad peak in higher temperature regions (strong basic sites). This profile indicated a wide distribution of basic sites. However, after loading of 3.0 wt% MgO, only weak basic sites remained preserved; the rest disappeared. Surprisingly, basic modifier addition caused the disappearance of basicity. XRD of the same sample showed the appearance of NiO–MgO-solid solution as well as the rise of ZrO_2_ crystallinity. This means that after the addition of basic 3.0 wt% MgO, basic MgO was engaged in the nurture of NiO–MgO solid solution and supported the crystallinity, thus it caused the disappearance of basicity. It caused the removal of intermediate strength as well as strong strength basic sites from the surface. Again, at 5 wt% MgO loading, peak reappeared in the intermediate temperature region whereas it broadened in high-temperature regions. As the TGA profile of the spent catalyst did not show markable carbon deposition, it is interesting to observe the basic profile of the spent catalyst.

The CO_2_-TPD profile of fresh as well as spent 5Ni/3Mg–ZrO_2_ & 5Ni/5Mg–ZrO_2_ catalyst are shown in Fig. 3D and Figure S3 respectively. Figures 3D and S3 also include O_2_-TPO and “CO_2_-TPO followed by O_2_-TPO” of spent 5Ni/3Mg–ZrO_2_ and 5Ni/5Mg–ZrO_2_ catalysts, respectively. It is obvious from the fresh and spent CO_2_-TPD samples that there was a significant decrease in the intensity of basic sites after the reaction over the spent catalysts. However, unlike the fresh samples, the spent catalysts showed a small peak in CO_2_-TPD. Again, a consumption (negative) peak in O_2_-TPO of spent 5Ni/3Mg–ZrO_2_ and spent 5Ni/5Mg–ZrO_2_ catalyst samples were also seen at about the same temperature region. Interestingly, O_2_-TPO (carried out after CO_2_-TPD) of spent 5Ni/3Mg–ZrO_2_ and spent 5Ni/5Mg–ZrO_2_ catalysts had no such O_2_ consumption peak. It can be explained that O_2_ consumption peak in O_2_-TPO was due to oxidation of residual carbon by O_2_ into CO_2_. So, the small evolution peak in CO_2_-TPD profile also indicated the oxidation of residual carbon deposit by CO_2_. As the carbon deposit on the surface of the catalyst was already oxidized by CO_2_ during CO_2_-TPD profile so when O_2_-TPO was carried out after CO_2_-TPD, no evolution peak was found. This confirmed the oxidation of the carbon deposit by CO_2_ over the surface of the catalyst^45,55^. Oxidation of carbon deposit by lattice oxygen of ZrO_2_ and thereafter simultaneous compensation of the oxygen vacant sites by CO_2_ (through losing one of its oxygen to the vacant site) might be a possible route of oxidation of carbon deposit by CO_2_.

**Figure 4:**
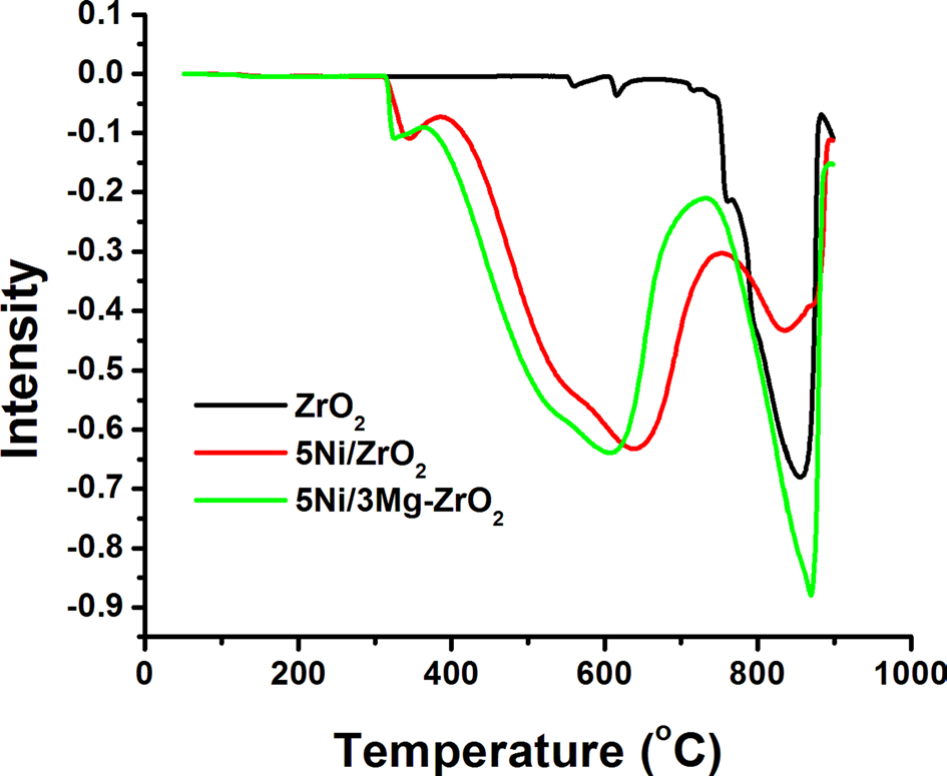
CH_4_-TPSR profile of catalysts.

To study the conditions and sites of CH_4_ decomposition, CH_4_-temperature programmed surface reaction (CH_4_-TPSR) experiment over ZrO_2_, 5Ni/ZrO_2_ and 5Ni/3Mg–ZrO_2_ were carried out (Fig. 4). It shows a decrease in the methane concentration with temperature over catalysts due to methane decomposition reaction on the surface. For ZrO_2_, a single prominent consumption peak at 870 °C temperature was noticed due to CH_4_ interaction at ZrO_2_ surface^53^. After the addition of Ni, apart from the high-temperature peak, a lower temperature CH_4_ consumption peak at about 350 °C and an intermediate temperature broad peak in the range of 400–800 °C were observed. Low temperature and intermediate temperature peaks could be claimed to the catalytic decomposition of CH_4_ over Ni active sites as well as Ni–Zr interface^53^. MgO containing catalysts (i.e. 5Ni/3Mg–ZrO_2_) also showed the intense peak at high temperature (about 800 °C), attributed to the effect of the temperature. At higher reaction temperature (about 800 °C), an endothermic feature of DRM reaction promotes more efficient catalytic decomposition of CH_4_ over Ni and Ni–Zr interface over 5Ni/3Mg–ZrO_2_ catalyst systems. This could explain the excellent CH_4_ conversion over the magnesium modified catalyst system. It is worth noting that the high-temperature peak is near to the reaction temperature region according to the CH_4_-TPSR profiles. That means if dry reforming of methane was carried out in the temperature region of 700 °C, an advantage of high temperature favourable endothermic feature (about 800 °C) of DRM reaction would be missing as shown in Fig. 4. It might be an indication of lower catalytic conversion at the lower reaction temperature.

### Discussion

**Figure 5:**
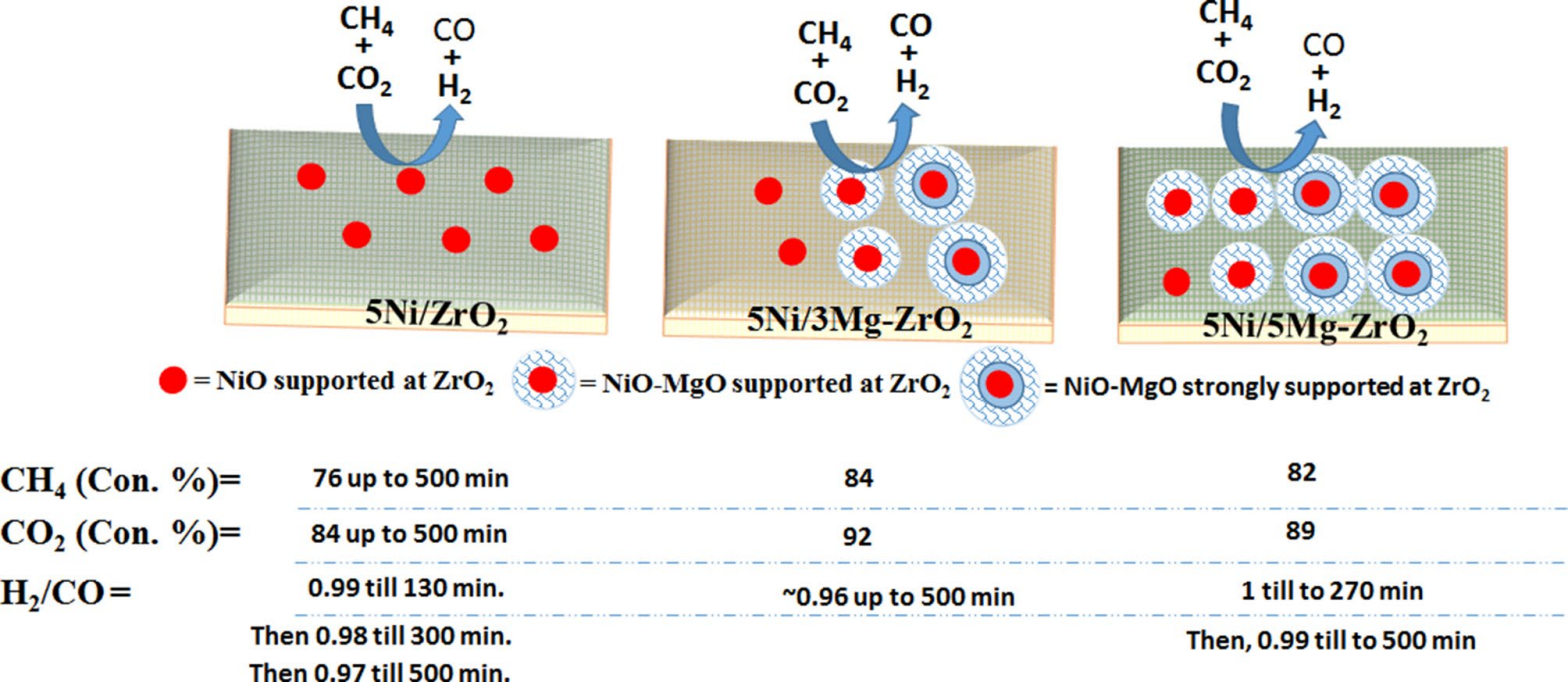
Summary of catalytic activity for different catalyst systems.

Thermal decomposition of CH_4_ and thereby oxidation of carbon deposits by CO_2_ towards dry reforming of methane is albeit possible with little activity i.e. 1.6% CH_4_ conversion, 3.6% CO_2_ conversion, H_2_/CO = 0.14. So, the catalytic role is utmost demanded in DRM. The summary of the catalytic activity of different catalysts towards dry reforming of methane is shown in Fig. 5. At 700 °C reaction temperature, comparable CH_4_ conversion, and CO_2_ conversion, were observed. At high reaction temperature, about 800 °C, an endothermic feature of DRM reaction was ruled over. It efficiently promotes catalytic decomposition of CH_4_ over Ni and Ni–Zr interface and thereafter oxidation of deposit by CO_2_. So, at 800 °C, all catalysts showed high CH_4_ and CO_2_ conversion as well as nearly no carbon deposit over the surface of the catalysts. Yang et al.^60^ also claimed MgO modified Ni system as outstanding coking tolerance and Chunwen et al.^61^ explained the effective reduction of carbon deposit by MgO modified Ni system by stabilization of lattice oxygen sites of NiO by MgO.

5Ni/ZrO_2_ had free NiO species, “NiO species interacted with support” and a wide range of basicity. CO_2_ uptake at basic sites, catalytic decomposition of CH_4_ at Ni and Ni–Zr and oxidation of deposits by CO_2_ pivoted the way of high-performance dry reforming reaction. It showed a constant 76% CH_4_ conversion, constant 84% CO_2_ conversion and 0.99 H_2_/CO ratios for 130 min, then a ratio of 0.98 for 300 min and finally a ratio of 0.96 for 500 min.

After modifying the catalyst with 3.0 wt% MgO, NiO–MgO-solid solution was built up. With a wide range of NiO–MgO-solid solution interaction (weakly as well as strongly with support ZrO_2_), 5Ni/3 Mg–ZrO_2_ promoted the efficient catalytic decomposition of CH_4_ over Ni, Ni–Zr interface and thereafter oxidation of deposit by CO_2_**.** Thus, 5Ni/3Mg–ZrO_2_ showed high 85% CH_4_ conversion and 92% CO_2_ conversion with H_2_/CO ratio ~ 0.96. The CO_2_-TPD, as well as O_2_-TPO profile of spent catalysts, showed an extra peak in TPD and a negative (consuming) peak in TPO, respectively which both related to the oxidation of residual carbon deposits on the surface of the catalyst. The CO_2_-TPD along with the O_2_-TPO results showed that CO_2_ is capable of oxidizing carbon deposit over the surface of the catalyst. Removal of carbon deposits by hydrogen gas through methane gasification reaction (C + 2H_2_ → CH_4_) is also possible^64^. It resulted in stable catalytic activity up to 500 min in the TOS test. Furthermore, modifying the catalyst with 5 wt%MgO in 5Ni/5Mg–ZrO_2_, it showed more amount of “NiO–MgO solid solution strongly interacted with ZrO_2_ support” as well as a wide variety of basic sites. That catalyst showed a constant conversion (82% CH_4_ conversion and 89% CO_2_ conversion) as well as H_2_/CO ratio = 1 for 250 min in the TOS then slightly decreased to 0.99 for another 250 min, with overall 500 min TOS. Thus, it could be concluded that 5 wt% MgO loading is optimum loading for an active and stable catalyst for methane dry reforming reaction. Further increase in magnesium oxide loading to 7 wt% MgO caused a decrease in NiO–MgO-solid solution that interacted weakly or strongly with the ZrO_2_ support and consequently the loss of strong basic sites. Thus, decreasing the CH_4_ conversion to 79% as well as CO_2_ conversion to 86% and H_2_/CO ratio to 0.98 were noticed.

## Conclusion

Magnesium promoted NiO supported mesoporous zirconia, 5Ni/xMg–ZrO_2_ (x = 0, 3, 5, 7) were prepared and tested for the methane dry reforming reaction. Higher activity was found at 800 °C than that at 700 °C due to favourable endothermic feature of DRM reaction which promotes efficient CH_4_ decomposition over Ni and Ni–Zr interface and successive oxidation of carbon deposits by CO_2_. By modifying the catalyst (5Ni/ZrO_2_) with MgO as a promoter, NiO–MgO-solid solution was formed. It was found that for high constant CH_4_ and CO_2_ conversions, NiO–MgO-solid solution played a significant role during the DRM. The 5Ni/3Mg–ZrO_2_ catalyst showed a constant 85% CH_4_ conversion and 92% CO_2_ conversion up to 500 min on stream at H_2_/CO mole ratio ~ 0.96. The highly constant performance of magnesium oxide modified catalysts was due to the ability of CO_2_ to oxidize the carbon deposits during the DRM, thus maintaining the catalytic stability. However, with a further loading (> 5.0 wt% Mg) such as in 5Ni/5Mg–ZrO_2_ which showed a higher amount of “NiO–MgO-solid solution strongly interacted with ZrO_2_ support” along with a wide variety of basic sites as well. Thus, it showed a constant 82% CH_4_ conversion and 89% CO_2_ conversion and H_2_/CO mole ratio ~ 1. It is hoped that these findings could inspire finding more stable and less expensive synthesis gas production catalysts, including from two potent greenhouse gases emissions such as methane and carbon dioxide.

## Experimental

### Materials

Nickel nitrate hexahydrate [Ni (NO_3_)_2_.6H_2_O, 98%, Alfa Aesar], magnesium acetate tetra-hydrate [Mg(O_2_CCH_3_)_2_.4H_2_O, 99.5–102.0%, Merck], mesoporous zirconia (*meso*-ZrO_2_, 1/8" pellets, Alfa Aesar) were commercially available and were used without further purification. Ultrapure water was acquired from a Milli-Q water purification system (Millipore).

### Catalyst preparation

A two-step procedure, based on incipient wetness impregnation as described elsewhere^21^, was followed for synthesizing the desired catalysts. The first step was to dope the support with a metal oxide promoter, while the second step was to load nickel oxide over the promoted support. The detailed description of each synthesis step is given below.

### Synthesis of mesoporous zirconia promoted with magnesia (MgO-meso-ZrO_2_)

The required amount of Mg (CH_3-_CO_2_)_2_.4H_2_O for 3.0, 5.0, or 7.0 wt/wt% loading of MgO was mixed and pulverized with the required amount of *meso*-ZrO_2_. To this resultant solid mixture, drops of ultrapure water were added until the formation of a colourless paste, which was mechanically stirred until complete dryness at room temperature. The addition of water and drying processes were performed three times to ensure homogeneous distribution of Mg (CH_3_CO_2_)_2_ within the matrix of *meso*-ZrO_2_. The solid mixture was then grounded and calcined in a muffle furnace, at 600 °C for 3 h in the static air atmosphere. The resultant materials were designated as xMg–ZrO_2_ catalysts where x is wt% of MgO (x = 0, 3, 5, 7).

### Synthesis of mesoporous zirconia supported nickel oxide promoted with magnesia (NiO/MgO-meso-ZrO_2_)

The required amount of Ni (NO_3_)_2_.6H_2_O to obtain 5.0 wt/wt% of NiO loading was mixed and was crushed with the required amount of MgO-*meso*-ZrO_2_ of the desired MgO wt/wt% loading, forming a green solid mixture. Drops of ultrapure water were then added to get a paste. By continuous mechanical stirring, the paste was dried at room temperature. The wetting and drying processes were repeated three times. Afterwards, calcination was performed at 600 °C for 3 h in static air atmosphere. Overall, 5 wt% NiO loaded catalyst sample is designated as 5Ni/xMg–ZrO_2_ catalysts where x is wt% of MgO (x = 0, 3, 5, 7).

### Catalyst characterization

The details of instrument specifications and procedures are described in the supporting information and described elsewhere^21^.

### Catalyst test

DRM was carried out in a fixed-bed stainless steel tubular micro-reactor (ID = 9 mm) at atmospheric pressure. A load of 0.10 g catalyst was activated under 20 SCCM H_2_ flow at 800 °C for 60 min. Then 20 sccm of N_2_ was fed to the reactor for 20 min at 800 °C to remove adsorbed H_2_. Afterwards, CH_4_, CO_2_, and N_2_ were dosed at flow rates of 30, 30 and 5 sccm, respectively. A GC (GC-2014 Shimadzu) unit, equipped with a thermal conductivity detector and two columns, Porapak Q and Molecular Sieve 5A, was connected in series/bypass connections to have a complete analysis of the reaction products. The following equations were used to calculate the conversion of each reactant and the H_2_/CO mole ratio, respectively^21^.

$$\begin{aligned} & {\text{CH}}_{4} \;{\text{conversion}} = { }\frac{{{\text{CH}}_{{4,{\text{in}}}} - {\text{CH}}_{{4,{\text{out}}}} }}{{{\text{CH}}_{{4,{\text{in}}}} }}{ } \times { }100{\text{\% }} \\ & {\text{CO}}_{2} { }\;{\text{conversion}} = { }\frac{{{\text{CO}}_{{2,{\text{in}}}} - {\text{CO}}_{{2,{\text{out}}}} }}{{{\text{CO}}_{{2,{\text{in}}}} }}{ } \times { }100{\text{\% }} \\ & \frac{{{\text{H}}_{{2{ }}} }}{{{\text{CO}}}}{ } = { }\frac{{{\text{mole}}\;{\text{ of }}\;{\text{H}}_{2} \;{\text{produced}}}}{{{\text{mole }}\;{\text{of }}\;{\text{CO }}\;{\text{produced}}}} \\ \end{aligned}$$

## Supplementary information

Supplementary Information.
