# Supplementary material for: Promotional effect of magnesium oxide for a stable nickel-based catalyst in dry reforming of methane
Source: Sci Rep. 2020 Aug 17;10:13861. doi: 10.1038/s41598-020-70930-1 (PMC7431551; doi:10.1038/s41598-020-70930-1)
Supplement: Supplementary file 1 — Supplementary Information. [file 41598_2020_70930_MOESM1_ESM.docx]

*Supporting Information of*

**Promotional Effect of Magnesium Oxide for a Stable Nickel-based Catalyst in Dry Reforming of Methane**

Ahmed S. Al-Fatesh^1^*_,_ Rawesh Kumar^2^, Anis H. Fakeeha^1^, Samsudeen O. Kasim^1^, Jyoti Khatri^2^, Ahmed A. Ibrahim^1^, Rasheed Arasheed^3^, Muhamad Alabdulsalam^3^, Mahmud S. Lanre^1^, Ahmed I. Osman^4^***,** Ahmed E. Abasaeed^1^, Abdulaziz bagabas^3^

^1^Chemical Engineering Department, College of Engineering, King Saud University, P.O. Box 800, Riyadh 11421, Saudi Arabia

^2^Sankalchand Patel University, Visnagar, Gujarat, India, 384315

^3^National Petrochemical Technology Center (NPTC), King Abdulaziz City for Science and Technology, P.O. Box 6086, Riyadh 11442, Saudi Arabia

^4^School of Chemistry and Chemical Engineering, Queen’s University Belfast, Belfast BT9 5AG, Northern Ireland, UK

^*^Correspondence: [aosmanahmed01@qub.ac.uk](mailto:aosmanahmed01@qub.ac.uk) (A.I.O) Tel.: +44 2890 97 4412

[aalfatesh@ksu.edu.sa](mailto:aalfatesh@ksu.edu.sa) (A.S.A.-F) Tel.: +966-11-467-6859

**S1. Instrument specifications using for different characterization:**

The characterization techniques as described elsewhere ^1^. X-ray powder diffraction (XRD) patterns for the samples were recorded on a Bruker D8 Advance XR diffractometer by using Cu Kα radiation source and a nickel filter, operated at 40 kV and 40 mA. Phases were identified by a search-match technique using X’Pert High Score Plus software with reference to the JCPDS database. The textural characteristics of the catalysts were studied by N_2_ adsorption-desorption isotherms, computed at −197 °C with a Micromeritics Tristar II 3020 porosity and surface area analyzer. In each test, 0.2–0.3 g of catalyst was taken. The samples were previously degassed at 300 °C for 3 h to expel from the catalyst surface undesired adsorbed gases, organics, and water vapour. BET technique was applied to obtain the surface areas, using N_2_ adsorption scale in the interval of 0.06–0.35 times the equilibrium pressure. The determination of Temperature programming reduction (TPR) was executed by Micromeritics Auto Chem II device. 70 milligrams of samples were charged into the TPR chamber and flushed with Ar at 150 °C for half an hour. Then the temperature of the samples was cooled to 25 °C. Lastly, the furnace temperature was set to 1000 ^o^C at a rate of 10 °C/min ramp in 40 mL/min flow rate of a H_2_/Ar mixture (10:90 vol.%). A TCD unit helped to find the consumption of hydrogen. The Micromeritics Autochem II apparatus, Micromeritics analyzer was used to perform the Temperature programmed desorption of carbon dioxide (CO_2_ -TPD). Helium gas stream of 30 mL/min was used to outgas 0.05g of catalyst at 600 °C for 60 min. Then the catalyst was brought to 50°C. After that CO_2_ stream was admitted for 1 h, and the catalyst was then flushed with helium to remove the physical attached CO_2_. The desorption-peak profile was registered while the temperature was varied at 10°C/min. The CO_2_ composition in the output stream was determined using a thermal conductivity detector. The peak areas gave the quantity of CO_2_ desorbed. The thermal gravimetric analysis (TGA) was carried out under air flow, using a TGA-15 SHIMADZU analyser, to determine the amount of carbon deposited on the catalyst surface after the reaction, where 10-15 mg of the spent catalyst was filled into a platinum pan of the thermo-balance. Then the temperature was increased to 1000 °C at a heating rate of 20 °C.min^-1^ and the weight reduction was determined. Experiment Procedure for CH_4_-Temperature programmed surface reaction (CH_4_-TPSR) experiment is carried out by pretreated of the sample in flowing 10% H_2_/ Ar for 1 h at 700 °C. Then, the sample was cooled to room temperature in flowing Ar. The 10% CH_4_/Ar (30 mL/min) mixture gas was introduced into a sample at a heating rate of 10 °C /min from 100 to 800 °C.


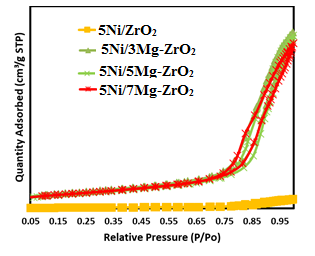


**Fig S1**. N_2_-physisorption isotherms of 5Ni/xMg-ZrO_2_ (x = 0,3,5,7) catalyst samples.





Fig. S2. H_2_-TPR of fresh and spent catalyst sample





Fig. S3. CO_2_-TPD and O_2_-TPO TPD profile of fresh and spent 5Ni/5Mg-ZrO_2_ catalyst.

**References**

[]. Fatesh, A. S. A., Kumar, R., Kasim, S. O., Ibrahim, A. A., Fakeeha, A. H., Abasaeed, A. E., Alrasheed, R., Bagabas, A., Chaudhary**,** M. L., Frusteri, F. & Chowdhury, B. The effect of modifier identity on the performance of Ni-based catalyst supported on γ-Al_2_O_3_ in dry reforming of methane. Catalysis today, **348**, 236-242 https://doi.org/10.1016/j.cattod.2019.09.003 (2020).
